# Evidence for the Role of Blue Light in the Development of Uveal Melanoma

**DOI:** 10.1155/2015/386986

**Published:** 2015-05-17

**Authors:** Patrick Logan, Miguel Bernabeu, Alberto Ferreira, Miguel N. Burnier

**Affiliations:** ^1^The Henry C. Witelson Ocular Pathology Laboratory, McGill University, Montreal, QC, Canada H3A 2B4; ^2^Alcon Laboratories Inc., Fort Worth, TX 76134, USA

## Abstract

Uveal melanoma is the most common malignancy of the adult eye. Although it is a relatively infrequent tumor, clinical prognosis is often poor owing to a high incidence of aggressive metastatic disease, for which there are limited treatment options. Little is known about the etiology of this condition, although several risk factors have been identified. Unlike cutaneous melanoma, however, ultraviolet radiation does not figure prominently among these risk factors. In this review, we focus on an associated form of visible electromagnetic radiation, high-energy short-wave (blue) light, a causative agent in various forms of age-related retina damage, as a previously overlooked risk factor in uveal melanoma development and progression. Finally, we discuss the impact of these data on contemporary ocular therapy, particularly the debate surrounding the filtering capabilities of intraocular lenses used to replace dysfunctional crystalline lenses during cataract surgery.

## 1. Introduction

Uveal melanoma (UM) is the second most common primary malignancy of the eye worldwide after childhood retinoblastoma and is the principal fatal intraocular disease in adults [1]. The reported incidence ranges from 4 to 11 cases per million/year [2–4], varying by country and ethnicity, with a significantly higher incidence in Caucasians than in African and Asian populations. UM arises in melanocytes located within the three different regions of the uveal tract: the iris, ciliary body, and choroid.

UM can be diagnosed at any age but it is more common in middle to later life, with a median age of onset of 55–60 years [1, 3]. Although it is a relatively rare neoplasm, UM is associated with particularly high mortality, primarily due to a high level of metastatic liver disease which develops in almost half of patients within 15 years of diagnosis [5, 6]. Once liver metastasis is detected, prognosis is poor, with a reported median survival duration ranging from 2.2 to 12.5 months [7–9]. A poor clinical outcome, coupled with limited treatment options (there are no specific therapeutic modalities currently available for metastatic UM) [10–12], underscores the need to define precisely the underlying risk/predisposing factors that lead to uveal melanocyte transformation and subsequent metastasis, with the focus as much on prevention as on early diagnosis.

Various risk factors associated with UM have been identified. A correlation between UM incidence and latitude in European populations suggests that lack of ocular pigmentation could be a risk factor for UM [4, 13, 14], and light iris color has also been linked to poor prognosis in patients with UM [15]. Within populations with light irises, however, elevated levels of choroidal pigmentation have been linked to an increased incidence of UM [16]. Other recognized risk factors include Caucasian ethnicity [13], preexisting choroidal nevi [17, 18], and genetic factors such as specific chromosomal abnormalities and* GNAQ/11* mutations [19, 20]. Furthermore, some familial factors have been associated with an increased risk of UM and the prevalence is higher in men than women [19].

A link between ultraviolet exposure and UM, as observed with cutaneous melanoma, has been suggested, but the evidence for this is inconclusive [21, 22]. It has been known for many years that ultraviolet exposure, coupled with specific skin pigment gene polymorphisms, is a prominent factor in the development of cutaneous melanoma [23, 24]. This strong link has in turn driven efforts to establish whether there is a similar link in patients with UM; however, epidemiological and genetic studies have generally failed to show such a connection. Sunlight exposure does not show a strong association with UM [21, 22], although arc welding (a source of ultraviolet/blue light) does [21, 22, 25].

Genetic studies also suggest that ultraviolet radiation does not significantly contribute to UM. For example, the oncogenic* V600E bRAF* mutation, expressed in the majority of patients with cutaneous melanoma, is thought to be the result of solar ultraviolet exposure [26–28] and is absent from melanomas occurring in body locations that are naturally protected from ultraviolet radiation [28]. This link provides a useful molecular tool that allows direct insight into the contribution of solar ultraviolet radiation to UM incidence [29, 30]. Genetic analysis of* V600E bRAF* expression in patients with UM has uncovered a relationship between the frequency of this mutation and the ocular location of the melanoma.* V600E* mutations have been detected in patients with anterior UM, such as those of the iris [31], consistent with ultraviolet exposure; however, most UM cases arise in the posterior uveal tract and* V600E* mutation rates here are negligible [19, 32]. These data imply that while ultraviolet radiation may well play a role in some cases of anterior UM, it does not significantly contribute to the oncogenic changes driving the majority of UM arising in the posterior uveal tract.

The combined epidemiological/genetic case against a significant role for ultraviolet radiation in the etiology of this disease is consistent with the established properties of the adult crystalline lens and cornea, which collectively filter out all wavelengths below 400 nm [29, 30, 33, 34]. Perhaps the key to understanding the link between UM and activities that generate high amounts of electromagnetic radiation (e.g., arc welding) does not lie in what is filtered out but in what can pass through the lens and cornea. For instance, arc welding produces significant amounts of intense short-wave light [25, 35]. Unlike ultraviolet radiation, short-wave (perceived as blue) light (400–500 nm) can reach the posterior uveal tract while retaining sufficient energy to be deleterious to biological structures. In fact, although visible light reaching the retina is a prerequisite for sight, phototoxic damage caused by its higher energy blue component is not an uncommon feature of the mammalian eye. A significant number of articles have documented blue-light-mediated damage to cells derived from the retinal pigment epithelium, retinal ganglion cell layer, and other epithelia, especially blue light in the 425–475 nm range [36–41]. This cellular damage is thought to be primarily photochemical and arises from chromophores, such as melanin, retinoids, and lipofuscin. Blue light can also generate reactive oxygen species (ROS) in mitochondria [36]. This damage usually results in cell dysfunction or death, the main causes of cellular aging and age-related macular degeneration (AMD), but may also contribute to tumorigenesis [41]. In this paper, we review the available evidence for a causal link between blue light and UM.

## 2. Role of Blue Light in Uveal Melanoma

Systematic literature searches conducted through Ovid (Embase and PubMed) showed that the earliest reports of an association between exposure to blue light and the development of UM have come from* in vitro* and animal work. Several studies have shown that blue light has a mitotic effect on human UM cell lines [42, 43]. Cultured human UM cells exposed to blue light (peak 475 nm) significantly increased their mitotic division rate relative to blue-light-shielded controls, an effect that was blocked using a blue-light-filtering lens [43]. Although the exact mechanism underlying the relationship between blue light and increased proliferation of uveal melanoma cells is unknown, it has been shown that shorter wavelengths of light can induce retinal pigment epithelial cell death by mitochondrial-derived ROS production [40]. Thus, although specific studies are required, investigating ROS production following blue light exposure in uveal melanoma cells would be a good starting point for elucidating the aforementioned relationship.

This interesting observation was followed up by a study that sought to mimic the effect of blue light on UM cells within the context of the mammalian eye. Human UM cells xenografted into the eye of an albino rabbit model of ocular melanoma and subsequently exposed to blue light showed enhanced proliferation upon removal and reculture, compared with control samples protected from blue light [42]. The significance of this finding is that the UM cells were exposed to blue light while residing within the choroid, effectively demonstrating that blue light affects uveal cells and can enhance their mitotic ability, a crucial step in linking blue light to malignant changes within uveal melanocytes* in vivo*. A final confirmation of the link between blue light and UM* in vivo* comes from a study in Long Evans rats, a strain with pigmented eyes in which there have been no reported cases of intraocular melanoma. This study described the development of an ocular tumor in one animal following blue light exposure (434–475 nm) coupled with the administration of an antiapoptotic agent [44]. The tumor involved the iris, ciliary body, choroid, and sclera, and contained large amounts of melanin.

Paradoxically, however, 450 nm blue light appears to be phototoxic to mouse cutaneous melanoma cells [45, 46]. Initial work reported that 450 nm light from a light-emitting diode was cytostatic to B16 cells, [45] as well as inhibiting the ability of these cells to metastasize to the lung when injected intravenously into mice [47]. Further work has extended these early observations to show that 450 nm light is both cytostatic and cytocidal to B16 cells [46]. It has also been suggested that blue light therapy may be of clinical benefit in cases of hemorrhagic metastatic melanoma, based on data from a single patient receiving aggressive chemotherapy for a melanoma on the anterior forearm. It should be noted that these phototoxic data are all based on cutaneous melanoma and for the most part the behavior of a single mouse cell line.

Further evidence underpinning a link between blue light and UM comes from neonatal blue light therapy studies. Blue light therapy is an essential tool in treating neonatal jaundice because dermal/subcutaneous bilirubin absorbs light maximally at 425–475 nm, leading to its conversion to a less toxic soluble form. A reported long-term side effect of this therapy is the increased risk of dysplastic nevus development in both the skin and eye (clinically, atypical nevi, or dysplastic nevi, are generally accepted to increase an individual's risk of melanoma) [48]. An initial report described a higher prevalence of atypical but not common melanocytic nevi in the skin of school children who had previously received neonatal blue light therapy [49]. Although broad-spectral emission bulbs are frequently used in this therapy (370–600 nm, maximal emission 450 nm), ultraviolet A contamination remains negligible at approximately 0.3% of output [48]. A follow-up study of monozygotic and heterozygotic twins, in which one of each pair had received and one had not received neonatal blue light therapy, not only reproduced these findings, but also found an increase in benign ocular pigmented lesions of the iris in the cohort who had received blue light therapy [50]. The latter finding is surprising because eye protection is worn in neonatal blue light therapy; however, accidental removal of eye coverings may occur and the neonatal eye allows greater transmission of lower frequency blue light relative to the adult eye [29].

Blue light has been shown to induce nuclear DNA lesions, suggesting a possible mechanism for tumorigenesis. Specific nuclear DNA lesions resulting from blue light have been recorded in the presence of lipofuscin, a photoinduced intracellular generator of ROS [36]. Furthermore, although no study has shown DNA mutations in uveal melanocytes in the presence of blue light, the carcinogenic potential of radiation in the 365–436 nm ultraviolet A/blue light crossover region has been demonstrated. In animal models of melanoma, ultraviolet A/blue light is not directly absorbed by DNA but rather exerts its effect through a photochemical interaction with melanin [51]. Given the filtering power of the crystalline lens and cornea, there is a significant window of mutagenic opportunity for blue light of 400–436 nm, and probably higher, because 365–436 nm represented the peak of activity. Interestingly, it has been shown that ultraviolet B and ultraviolet A differentially induce cutaneous melanoma through direct DNA damage and indirect melanin-derived ROS-mediated damage, respectively [52]. The wavelengths of ultraviolet A and blue light are adjacent and their biological effects probably overlap, as their shared ability to generate ROS demonstrates. Although the relationship between melanocyte melanin (eumelanin, pheomelanin, and their precursors), ROS generation, and DNA damage is complex [53], a plausible scenario involving blue-light-triggered ROS-induced melanocyte mutation can be hypothesized as a precipitating factor leading to UM.

Blue light may also play a role in neovascularization, a key event in the successful development of any solid tumor, and by extension UM. A central player in this process is vascular endothelial growth factor (VEGF), a signaling protein that stimulates both vasculogenesis and angiogenesis. Hypoxia is known to induce VEGF production by UM cells [54, 55], and high levels of VEGF have been reported in both the aqueous and vitreous humors of patients with UM [56, 57]. Furthermore, VEGF is an associated biomarker of UM metastasis [58, 59]. In cultured retinal pigment epithelial cells, VEGF production in response to white light exposure can be inhibited by directly blocking the blue light component [60–62]. While it remains to be seen if this phenomenon can be replicated in either primary ocular melanocytes or UM cells, particularly with respect to its relationship with hypoxia, these data lend further mechanistic credibility to the link between blue light and a critical stage in the development of UM.

## 3. Clinical Implications

**Figure 1:**
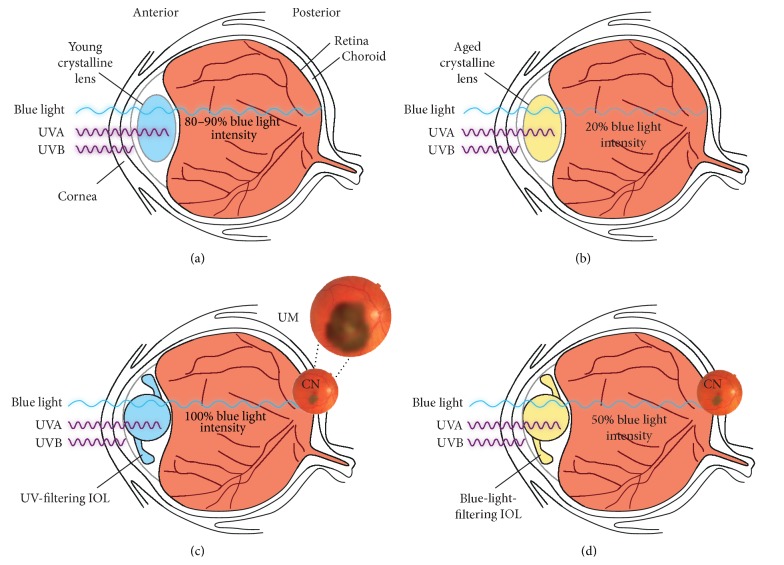
(a) The young crystalline lens and cornea together filter UVA and UVB while allowing transmission of most blue light (defined as 400–500 nm) to the retina. Around 80–90% of blue light at 450 nm can pass through the young lens. (b) As the crystalline lens ages it yellows and progressively filters more blue light until, by the sixth or seventh decade, blue light transmission can be as low as 20% of that transmitted by the young lens. (c) Early types of IOLs used to replace the crystalline lens during cataract surgery effectively filter UV but do not block blue light. It is hypothesized that blue light reaching the retina increases the risk of preexisting dysplastic nevi (indicated as CN, choroid nevus) progressing to UM. A typical CN is shown in the small retinal photograph, while a UM is shown in the magnified retinal photograph. (d) Blue-light-filtering IOLs are designed to filter up to 50% of blue light. This models the natural filtering ability of the middle-aged eye, reducing potentially damaging radiation while not impacting on vision. We argue that preexisting CNs (shown in the small retinal photograph) are less likely to progress to UMs in this environment. CN, choroidal nevus; IOL, intraocular lens; UM, uveal melanoma; UV, ultraviolet; UVA, ultraviolet A; UVB, ultraviolet B.

If blue light is a known risk factor for AMD and, at the very least, a suspect in UM development, can this knowledge be translated into some form of meaningful preventive measure? One area of immediate relevance is the current debate surrounding intraocular lens (IOL) implants used after cataract surgery [63–65]. The replacement of the crystalline lens by an IOL is a central component of this procedure. IOLs are designed to filter out ultraviolet radiation to protect the eye from damage. Early IOL models filtered out ultraviolet radiation, but more recently developed IOLs filter out a wider spectral range, including at least the more energetic parts of blue light because, ideally, the spectral emission characteristics of an IOL should mimic those of the lens that was removed. As the crystalline lens ages, it yellows and filters out significantly more blue light above 400 nm. This process appears to be caused by the progressive accumulation of yellow chromophore deposits, primarily 3-hydroxykynurenine glucoside derivatives [66], estimated to reduce the blue light transmission capacity of the crystalline lens by around 0.7–0.8%/year [67]. Consequently, the 400–500 nm transmission capacity of 80–90% associated with the lens of a healthy child or young adult [68, 69] is cumulatively reduced in later life, dropping to around 50% by the fifth decade and to as little as 25% or less at 70 years and over [68–72]. There is also evidence to suggest that retinal tissue becomes more sensitive to phototoxicity, possibly owing to increased lipofuscin concentration and impaired antioxidant activity [29, 65], concomitant with altered lens function. Therefore, there is a clinical rationale for considering the use of blue-filtering IOLs, and this is further supported by the evidence presented in this paper (Figure 1).

## 4. Conclusions

In summary, cumulative epidemiological and experimental evidence indicates that blue light is a credible risk factor for the development of UM. Additional studies are required to clarify the risk associated with blue light and the protective potential of blue-filtering IOLs following cataract surgery. As life expectancy continues to increase, individuals are expected to live longer after cataract surgery. Shielding individuals from the known harmful effects of blue light, a role normally performed by the aging crystalline lens, through the use of blue-filtering IOLs is of clinical benefit as a preventive measure against UM.

## References

[1] Papastefanou V. P., Cohen V. M. (2011). Uveal melanoma. *Journal of Skin Cancer*.

[2] Singh A. D., Topham A. (2003). Incidence of uveal melanoma in the United States: 1973–1997. *Ophthalmology*.

[3] Singh A. D., Turell M. E., Topham A. K. (2011). Uveal melanoma: trends in incidence, treatment, and survival. *Ophthalmology*.

[4] Virgili G., Gatta G., Ciccolallo L. (2007). Incidence of uveal melanoma in Europe. *Ophthalmology*.

[5] (1998). The Collaborative Ocular Melanoma Study (COMS) randomized trial of pre-enucleation radiation of large choroidal melanoma II: initial mortality findings. COMS report no. 10. *American Journal of Ophthalmology*.

[6] Hawkins B. S., Collaborative Ocular Melanoma Study Group (2004). The collaborative ocular melanoma study (COMS) randomized trial of pre-enucleation radiation of large choroidal melanoma: IV. Ten-year mortality findings and prognostic factors. COMS report number 24. *The American Journal of Ophthalmology*.

[7] Rietschel P., Panageas K. S., Hanlon C., Patel A., Abramson D. H., Chapman P. B. (2005). Variates of survival in metastatic uveal melanoma. *Journal of Clinical Oncology*.

[8] Rajpal S., Moore R., Karakousis C. P. (1983). Survival in metastatic ocular melanoma. *Cancer*.

[9] Cerbone L., van Ginderdeuren R., van den Oord J. (2014). Clinical presentation, pathological features and natural course of metastatic uveal melanoma, an orphan and commonly fatal disease. *Oncology*.

[10] Mavligit G. M., Charnsangavej C., Carrasco C. H., Patt Y. Z., Benjamin R. S., Wallace S. (1988). Regression of ocular melanoma metastatic to the liver after hepatic arterial chemoembolization with cisplatin and polyvinyl sponge. *Journal of the American Medical Association*.

[11] Frenkel S., Nir I., Hendler K. (2009). Long-term survival of uveal melanoma patients after surgery for liver metastases. *British Journal of Ophthalmology*.

[12] Mariani P., Piperno-Neumann S., Servois V. (2009). Surgical management of liver metastases from uveal melanoma: 16 years' experience at the Institut Curie. *European Journal of Surgical Oncology*.

[13] Egan K. M., Seddon J. M., Glynn R. J., Gragoudas E. S., Albert D. M. (1988). Epidemiologic aspects of uveal melanoma. *Survey of Ophthalmology*.

[14] Saornil M. A. (2004). Iris colour and uveal melanoma. *Canadian Journal of Ophthalmology*.

[15] Schmidt-Pokrzywniak A., Kalbitz S., Kuss O., Jöckel K.-H., Bornfeld N., Stang A. (2014). Assessment of the effect of iris colour and having children on 5-year risk of death after diagnosis of uveal melanoma: a follow-up study. *BMC Ophthalmology*.

[16] Harbour J. W., Brantley M. A., Hollingsworth H., Gordon M. (2004). Association between choroidal pigmentation and posterior uveal melanoma in a white population. *British Journal of Ophthalmology*.

[17] Hammer H., Oláh J., Tóth-Molnár E. (1996). Dysplastic nevi are a risk factor for uveal melanoma. *European Journal of Ophthalmology*.

[18] van Hees C. L. M., de Boer A., Jager M. J. (1994). Are atypical nevi a risk factor for uveal melanoma? A case-control study. *Journal of Investigative Dermatology*.

[19] Harbour J. W. (2012). The genetics of uveal melanoma: an emerging framework for targeted therapy. *Pigment Cell and Melanoma Research*.

[20] van Raamsdonk C. D., Bezrookove V., Green G. (2009). Frequent somatic mutations of GNAQ in uveal melanoma and blue naevi. *Nature*.

[21] Shah C. P., Weis E., Lajous M., Shields J. A., Shields C. L. (2005). Intermittent and chronic ultraviolet light exposure and uveal melanoma: a meta-analysis. *Ophthalmology*.

[22] Singh A. D., Rennie I. G., Seregard S., Giblin M., McKenzie J. (2004). Sunlight exposure and pathogenesis of uveal melanoma. *Survey of Ophthalmology*.

[23] Elwood J. M., Jopson J. (1997). Melanoma and sun exposure: an overview of published studies. *International Journal of Cancer*.

[24] Abdel-Malek Z. A., Swope V. B., Starner R. J., Koikov L., Cassidy P., Leachman S. (2014). Melanocortins and the melanocortin 1 receptor, moving translationally towards melanoma prevention. *Archives of Biochemistry and Biophysics*.

[25] Fernandes B. F., Marshall J. C., Burnier M. N. (2006). Blue light exposure and uveal melanoma. *Ophthalmology*.

[26] Besaratinia A., Pfeifer G. P. (2008). Sunlight ultraviolet irradiation and BRAF V600 mutagenesis in human melanoma. *Human Mutation*.

[27] Pfeifer G. P., Besaratinia A. (2012). UV wavelength-dependent DNA damage and human non-melanoma and melanoma skin cancer. *Photochemical & Photobiological Sciences*.

[28] Cohen Y., Rosenbaum E., Begum S. (2004). Exon 15 BRAF mutations are uncommon in melanomas arising in nonsun-exposed sites. *Clinical Cancer Research*.

[29] Algvere P. V., Marshall J., Seregard S. (2006). Age-related maculopathy and the impact of blue light hazard. *Acta Ophthalmologica Scandinavica*.

[30] Mainster M. A., Turner P. L. (2010). Ultraviolet-B phototoxicity and hypothetical photomelanomagenesis: intraocular and crystalline lens photoprotection. *American Journal of Ophthalmology*.

[31] Henriquez F., Janssen C., Kemp E. G., Roberts F. (2007). The T1799A BRAF mutation is present in iris melanoma. *Investigative Ophthalmology and Visual Science*.

[32] Janssen C. S., Sibbett R., Henriquez F. L., McKay I. C., Kemp E. G., Roberts F. (2008). The T1799A point mutation is present in posterior uveal melanoma. *British Journal of Cancer*.

[33] Schwartz L. H., Ferrand R., Boelle P. Y., Maylin C., D'Hermies F., Virmont J. (1997). Lack of correlation between the location of choroidal melanoma and ultraviolet-radiation dose distribution. *Radiation Research*.

[34] Laube T., Apel H., Koch H.-R. (2004). Ultraviolet radiation absorption of intraocular lenses. *Ophthalmology*.

[35] Okuno T., Saito H., Ojima J. (2002). Evaluation of blue-light hazards from various light sources. *Developments in Ophthalmology*.

[36] Godley B. F., Shamsi F. A., Liang F. Q., Jarrett S. G., Davies S., Boulton M. (2005). Blue light induces mitochondrial DNA damage and free radical production in epithelial cells. *The Journal of Biological Chemistry*.

[37] Wood J. P. M., Lascaratos G., Bron A. J., Osborne N. N. (2008). The influence of visible light exposure on cultured RGC-5 cells. *Molecular Vision*.

[38] Roechlecke C., Schaller A., Knels L., Funk R. H. W. (2009). The influence of sublethal blue light exposure on human RPE cells. *Molecular Vision*.

[39] Kuse Y., Ogawa K., Tsuruma K., Shimazawa M., Hara H. (2014). Damage of photoreceptor-derived cells in culture induced by light emitting diode-derived blue light. *Scientific Reports*.

[40] King A., Gottlieb E., Brooks D. G., Murphy M. P., Dunaief J. L. (2004). Mitochondria-derived reactive oxygen species mediate blue light-induced death of retinal pigment epithelial cells. *Photochemistry and Photobiology*.

[41] Beatty S., Koh H.-H., Phil M., Henson D., Boulton M. (2000). The role of oxidative stress in the pathogenesis of age-related macular degeneration. *Survey of Ophthalmology*.

[42] Di Cesare S., Maloney S., Fernandes B. F. (2009). The effect of blue light exposure in an ocular melanoma animal model. *Journal of Experimental and Clinical Cancer Research*.

[43] Marshall J.-C. A., Gordon K. D., McCauley C. S., de Souza Filho J. P., Burnier M. N. (2006). The effect of blue light exposure and use of intraocular lenses on human uveal melanoma cell lines. *Melanoma Research*.

[44] Manning W. S., Greenlee P. G., Norton J. N. (2004). Ocular melanoma in a long evans rat. *Contemporary Topics in Laboratory Animal Science*.

[45] Ohara M., Kawashima Y., Katoh O., Watanabe H. (2002). Blue light inhibits the growth of B16 melanoma cells. *Japanese Journal of Cancer Research*.

[46] Sparsa A., Faucher K., Sol V. (2010). Blue light is phototoxic for B16F10 murine melanoma and bovine endothelial cell lines by direct cytocidal effect. *Anticancer Research*.

[47] Ohara M., Kawashima Y., Kitajima S., Mitsuoka C., Watanabe H. (2002). Inhibition of lung metastasis of B16 melanoma cells exposed to blue light in mice. *International Journal of Molecular Medicine*.

[48] Oláh J., Tóth-Molnár E., Kemény L., Csoma Z. (2013). Long-term hazards of neonatal blue-light phototherapy. *British Journal of Dermatology*.

[49] Csoma Z., Hencz P., Orvos H. (2007). Neonatal blue-light phototherapy could increase the risk of dysplastic nevus development. *Pediatrics*.

[50] Csoma Z., Tóth-Molnár E., Balogh K. (2011). Neonatal blue light phototherapy and melanocytic nevi: a twin study. *Pediatrics*.

[51] Setlow R. B., Grist E., Thompson K., Woodhead A. D. (1993). Wavelengths effective in induction of malignant melanoma. *Proceedings of the National Academy of Sciences of the United States of America*.

[52] Noonan F. P., Zaidi M. R., Wolnicka-Glubisz A. (2012). Melanoma induction by ultraviolet A but not ultraviolet B radiation requires melanin pigment. *Nature Communications*.

[53] Denat L., Kadekaro A. L., Marrot L., Leachman S. A., Abdel-Malek Z. A. (2014). Melanocytes as instigators and victims of oxidative stress. *Journal of Investigative Dermatology*.

[54] El Filali M., Missotten G. S. O. A., Maat W. (2010). Regulation of VEGF-A in uveal melanoma. *Investigative Ophthalmology and Visual Science*.

[55] Asnaghi L., Lin M. H., Lim K. S. (2014). Hypoxia promotes uveal melanoma invasion through enhanced Notch and MAPK activation. *PLoS ONE*.

[56] Boyd S. R., Tan D., Bunce C. (2002). Vascular endothelial growth factor is elevated in ocular fluids of eyes harbouring uveal melanoma: identification of a potential therapeutic window. *British Journal of Ophthalmology*.

[57] Missotten G. S. O., Notting I. C., Schlingemann R. O. (2006). Vascular endothelial growth factor A in eyes with uveal melanoma. *Archives of Ophthalmology*.

[58] Crosby M. B., Yang H., Gao W., Zhang L., Grossniklaus H. E. (2011). Serum vascular endothelial growth factor (VEGF) levels correlate with number and location of micrometastases in a murine model of uveal melanoma. *British Journal of Ophthalmology*.

[59] Barak V., Pe'er J., Kalickman I., Frenkel S. (2011). VEGF as a biomarker for metastatic uveal melanoma in humans. *Current Eye Research*.

[60] Yanagi Y., Inoue Y., Iriyama A., Jang W.-D. (2006). Effects of yellow intraocular lenses on light-induced upregulation of vascular endothelial growth factor. *Journal of Cataract and Refractive Surgery*.

[61] Kernt M., Neubauer A. S., Liegl R. (2009). Cytoprotective effects of a blue light-filtering intraocular lens on human retinal pigment epithelium by reducing phototoxic effects on vascular endothelial growth factor-*α*, Bax, and Bcl-2 expression. *Journal of Cataract and Refractive Surgery*.

[62] Hui S., Yi L., Fengling Q. L. (2009). Effects of light exposure and use of intraocular lens on retinal pigment epithelial cells in vitro. *Photochemistry and Photobiology*.

[63] Henderson B. A., Grimes K. J. (2010). Blue-blocking IOLs: a complete review of the literature. *Survey of Ophthalmology*.

[64] Mainster M. A., Turner P. L. (2010). Blue-blocking IOLs decrease photoreception without providing significant photoprotection. *Survey of Ophthalmology*.

[65] Edwards K. H., Gibson G. A. (2010). Intraocular lens short wavelength light filtering. *Clinical and Experimental Optometry*.

[66] Hood B. D., Garner B., Truscott R. J. W. (1999). Human lens coloration and aging. Evidence for crystallin modification by the major ultraviolet filter, 3-hydroxy-kynurenine O-*β*-D-glucoside. *Journal of Biological Chemistry*.

[67] Broendsted A. E., Hansen M. S., Lund-Andersen H., Sander B., Kessel L. (2011). Human lens transmission of blue light: a comparison of autofluorescence-based and direct spectral transmission determination. *Ophthalmic Research*.

[68] Boettner E. A., Wolter J. R. (1962). Transmission of the ocular media. *Investigative Ophthalmology & Visual Science*.

[69] Artigas J. M., Felipe A., Navea A., Fandiño A., Artigas C. (2012). Spectral transmission of the human crystalline lens in adult and elderly persons: Color and total transmission of visible light. *Investigative Ophthalmology and Visual Science*.

[70] Kessel L., Lundeman J. H., Herbst K., Andersen T. V., Larsen M. (2010). Age-related changes in the transmission properties of the human lens and their relevance to circadian entrainment. *Journal of Cataract and Refractive Surgery*.

[71] Dillon J., Zheng L., Merriam J. C., Gaillard E. R. (2004). Transmission of light to the aging human retina: possible implications for age related macular degeneration. *Experimental Eye Research*.

[72] Ernest P. H. (2004). Light-transmission-spectrum comparison of foldable intraocular lenses. *Journal of Cataract and Refractive Surgery*.

